# The effects of maximising the UK’s tobacco control score on inequalities in smoking prevalence and premature coronary heart disease mortality: a modelling study

**DOI:** 10.1186/s12889-016-2962-8

**Published:** 2016-04-01

**Authors:** Kirk Allen, Chris Kypridemos, Lirije Hyseni, Anna B. Gilmore, Peter Diggle, Margaret Whitehead, Simon Capewell, Martin O’Flaherty

**Affiliations:** Lancaster Medical School, Lancaster University, Lancaster, UK; Department of Public Health & Policy, University of Liverpool, Liverpool, UK; Department for Health, University of Bath, UK Centre for Tobacco and Alcohol Studies (UKCTAS), Bath, UK

**Keywords:** Tobacco control, Framework Convention on Tobacco Control (FCTC), Coronary heart disease, Socioeconomic inequalities

## Abstract

**Background:**

Smoking is more than twice as common among the most disadvantaged socioeconomic groups in England compared to the most affluent and is a major contributor to health-related inequalities. The United Kingdom (UK) has comprehensive smoking policies in place: regular tax increases; public information campaigns; on-pack pictorial health warnings; advertising bans; cessation; and smoke-free areas. This is confirmed from its high Tobacco Control Scale (TCS) score, an expert-developed instrument for assessing the strength of tobacco control policies. However, room remains for improvement in tobacco control policies.

Our aim was to evaluate the cumulative effect on smoking prevalence of improving all TCS components in England, stratified by socioeconomic circumstance.

**Methods:**

Effect sizes and socioeconomic gradients for all six types of smoking policy in the UK setting were adapted from systematic reviews, or if not available, from primary studies.

We used the IMPACT Policy Model to link predicted changes in smoking prevalence to changes in premature coronary heart disease (CHD) mortality for ages 35–74. Health outcomes with a time horizon of 2025 were stratified by quintiles of socioeconomic circumstance.

**Results:**

The model estimated that improving all smoking policies to achieve a maximum score on the TCS might reduce smoking prevalence in England by 3 % (95 % Confidence Interval (CI): 1–4 %), from 20 to 17 % in absolute terms, or by 15 % in relative terms (95 % CI: 7–21 %). The most deprived quintile would benefit more, with absolute reductions from 31 to 25 %, or a 6 % reduction (95 % CI: 2–7 %).

There would be some 3300 (95 % CI: 2200–4700) fewer premature CHD deaths between 2015–2025, a 2 % (95 % CI: 1.4–2.9 %) reduction. The most disadvantaged quintile would benefit more, reducing absolute inequality of CHD mortality by about 4 % (95 % CI: 3–9 %).

**Conclusions:**

Further, feasible improvements in tobacco control policy could substantially improve population health, and reduce health-related inequalities in England.

**Electronic supplementary material:**

The online version of this article (doi:10.1186/s12889-016-2962-8) contains supplementary material, which is available to authorized users.

## Background

Smoking remains common in England, with 19 % of adults aged 16 and over reported as smokers in 2013 [1]. This prevalence changed slightly from 21 % in 2007 [2]. Furthermore, large differences in smoking prevalence persist across socioeconomic groups; over 30 % of people with routine and manual jobs smoke, compared to less than 15 % of those in managerial and professional occupations [2, 3]. Smoking explains more than one-quarter of the socioeconomic gradient in total mortality in Great Britain [4].

In 2013, smoking caused an estimated 80,000 deaths in England among adults aged 35 and over. This amounts to 17 % of all deaths for these ages, unchanged since 2005. Over 450,000 hospital admissions were attributable to smoking, representing 4 % of all adult admissions [5]. These numbers may underestimate the true burden of smoking, as a recent study has expanded the list of diseases linked to smoking [6].

Circulatory diseases alone represent 17,300 (13 %) deaths and 134,000 (19 %) hospital admissions [2]. Coronary heart disease (CHD) has the highest number of smoking attributable deaths (7900) among circulatory diseases and the third highest number overall, after lung cancer and chronic obstructive pulmonary disease [2].

The United Kingdom (UK) has strong tobacco control policies compared to many European peers, achieving the highest score on the Tobacco Control Scale (TCS) (74 out of 100) among 34 European countries [7]. The TCS is an expert-developed instrument for assessing the strength of tobacco control policies with data compiled via a survey of national representatives to the European Network for Smoking and Tobacco Prevention, supplemented with data from other data sources (described in more detail in [7]). The most recent survey took place in 2013 and represents legislation in place as of 1 January 2014, prices as of 1 July 2013, and tobacco control budget for 2012. The TCS assesses six types of tobacco control policies; price, public place bans, public information campaign spending, advertising bans, health warnings, and treatment, each based on several indicators. World Health Organization MPOWER rankings for the UK are reassuringly similar [8].

Systematic reviews have found some evidence of socioeconomic gradients in effectiveness of tobacco control policies [9–12]. However, with the exception of taxation, this evidence is limited, mixed and further complicated by different definitions of socioeconomic circumstance (e.g. education, occupation, income). Interestingly, evidence suggest that tobacco tax increases are more effective among the less well-off.

There is a gap in the literature relating socioeconomic inequalities in effectiveness of tobacco control policies, to inequalities in health outcomes. In this study, we therefore evaluated the potential effectiveness of maximising the TCS score for the UK using a model stratified by socioeconomic circumstance (SEC). We then linked predicted reductions in smoking prevalence to reductions in premature CHD mortality in England and assessed changes in inequality of premature CHD mortality.

## Methods

The previously validated, deterministic, cell-based IMPACT Policy Model has been used to model the change in adult smoking prevalence, in England, that might result from changes in tobacco control policies, and translated it to CHD deaths using a population attributable risk fraction approach [13, 14]. The method has been previously used to analyse health benefits of reduced smoking prevalence in other European countries [15]. The uncertainty was estimated using probabilistic sensitivity analysis in a Monte Carlo approach (please refer to the Additional file 1 for a detailed description). Of the four UK countries, analysis was restricted to England because smoking prevalence can be directly linked to a health outcome using a socioeconomic indicator.

### Smoking prevalence

Smoking prevalence in 2012 by age, sex, and quintile groups of Index of Multiple Deprivation (IMDQ) for England were published by the Office for National Statistics (ONS). ONS estimates were extracted from the 2012 Integrated Household Survey, which contains information from approximately 340,000 individual respondents [16]. We used this smoking prevalence from 2012 as the baseline from which reductions could occur. Age was summarized into groups: 18–24, 25–34, 35–44, 45–54, 55–64, 65–74, and 75+. We used only the age groups 35–44, 45–54, 55–64, and 65–74 so as to capture premature adult CHD deaths. IMDQ is an area-based socioeconomic indicator composed of seven domains of deprivation (income, employment, health, education, crime, access to services, living environment), each with around five indicators [17].

### Policy scenario

We modelled an increase in each component of the TCS to raise the overall score to the perceived maximum of 100. This would bring the UK fully in line with currently accepted best practices.

Changes in smoking prevalence due to policy changes were adapted primarily from systematic reviews. When these were not available, we used relevant primary studies or inputs used in published modelling studies. Table 1 shows the policy types, current UK status, maximum effect sizes, SEC gradients, and modelling decisions:

**Table 1:** UK’s status of tobacco control policies and additional modelled policies to maximise Tobacco Control Scale

Policy type	UK status (2013) [Additional modelled policies]	Maximum effect on smoking prevalence	SEC gradient	Model decision
Price	27 out of 30 [20 % retail price increase]	3.5 % reduction for 10 % price increase [19]	For each 10 % price increase, prevalence relative decreases by [18]: Lowest SEC: 6.3 %	20 % price increase. The effect on prevalence was modelled from published price elasticities by SEC.
			Highest SEC: 1.2 %	
Smoke-free places	21 out of 22 [Smoking in cars with minors banned as of October 2015 and extend ban to all public places]	Worksite total ban 6 % reduction compared to 2 % for partial ban; Restaurant total ban 1 % reduction [21, 22]	Smoke-free workplaces generally favour higher SEC [9, 12]. Mixed evidence for other types smoke-free places [10, 12].	Additional 1 % prevalence relative reduction possible because little room for improvement. Assume no SEC gradient.
Public information campaigns	3 out of 15 [a five-fold increase to 2012 government budget spending media campaigns]	Maximum annual effect 2 % [26, 27]	Often favour highest SEC [28]	Additional 1 % (average) prevalence relative reduction possible because moderate campaigns already in place. Assume Highest SEC twice as responsive as Lowest SEC.
Advertising bans	10 out of 13 [Point-of-sale and display ad ban in small stores as of April 2015]	Comprehensive ban 5 % prevalence reduction; Total ban 3 % reduction; Weak ban 1 % reduction [21, 29]	No evidence of gradient [9, 10]	Additional 2 % prevalence relative reduction possible
Health Warnings (including plain packaging)	4 out of 10 [Plain packaging approved by Parliament, larger health warnings (>80 % of the packet)]	Large bold graphic warnings reduce prevalence by 2 %; Weaker warnings 1 % reduction. Plain packaging has maximum effect similar to health warnings [33]	No evidence of gradient [9, 10, 35]	Additional 3 % prevalence relative reduction possible (1 % from larger health warnings and 2 % from plain packaging).
Treatment	9 out of 10 [Full reimbursement of treatment]	4.75 % reduction in prevalence (no details on individual components of treatment policy) [21]	Low SEC may have lower success, but programs can be targeted to eliminate gradient [36]	Additional 0.5 % prevalence relative reduction possible because most elements in place already. No SEC gradient
SEC denotes Socioeconomic circumstance				
UK status for 2013 (2^nd^ column) is based on Tobacco Control Scale [7]				

Effect on prevalence, socioeconomic gradient, and parameters used in model for changes in policies. Uncertainty in the policy effect sizes is described in Additional file 1: Table S1

#### Price

TCS ranks average price per pack in the UK as the highest in Europe. We modelled a further 20 % increase in retail price, equivalent to an increase in excise duty from approximately 61 to 81 % of the retail price. The relationship between tax increases and smoking prevalence (i.e. the elasticities) were based on the British setting, including a socioeconomic gradient that makes price increases more effective among those of lower status [18, 19] (Additional file 1: Table S2). Data from the United States (US), Australia and Canada suggest the same relationship between elasticity and socioeconomic status [20].

#### Smoke-free public places

The UK scores highly on smoke-free places with the top score for bars & restaurants, public transport, and work places. There remains room for improvement in other public places (e.g. education, health). The relationship between smoking prevalence and smoke-free places was based on inputs to other modelling studies [21, 22]. A systematic review of workplace bans reported higher effectiveness among those to whom the ban applied [23], but the population effect would be lower. We assumed a small additional benefit could be achieved from further bans in public places not currently covered. We assumed no SEC gradient because there is mixed evidence [10, 12]. The evidence more strongly supports SEC gradients that benefit the more affluent for workplace bans [9, 12]; however, the UK already has strong workplace bans.

#### Public information campaigns (mass media)

Anti-tobacco campaign spending was the TCS component with the most room for improvement due to funding cuts in 2010 that resulted in large declines in quit-line calls, anti-smoking literature requests, and hits to the smoking cessation website [24]. Funding rebounded somewhat by 2012 [25]. We used the maximum single-year effect for public information campaigns on smoking prevalence [26, 27]. Then, we assumed that half of that maximum effect could be achieved after maximizing the TCS public information campaign component, because moderate campaigns are already in place. Although there is some controversy regarding the equity of these interventions, when cessation is considered as the outcome, their SEC gradient likely favours the more affluent [12, 28].

#### Advertising bans

The UK has advertising bans in most areas: TV/radio, cinema, outdoor, print, and sponsorship. Advertising bans at point of sale and displays were implemented in April 2015 but were not in the TCS score, nor would their effect be represented in the smoking prevalence data due to their recency. Comprehensive advertising bans could have a maximum effect on consumption of about 7 % [21, 29]. The maximum effect on prevalence would be about 5 % if we assume that 70 % of the reduction in consumption is due to a reduction in prevalence, as typically observed for price increases [19]. We modelled that the newly implemented components of advertising bans would result in a 2 % reduction in prevalence. There is no evidence of SEC gradients in response to advertising bans [10, 12]. Standardized packs are also a type of advertising ban in the TCS, and these are discussed more extensively below.

#### Health warnings (including plain packaging)

On-pack health warnings in the UK contain a pictorial warning, but they could be larger. The maximum effectiveness of health warnings was based on other modelling studies [21, 22], which is a fair midpoint between low estimates of the US Food and Drug administration (FDA) and high estimates from Canada (discussed in [30]). There is no evidence of a SEC gradient [9, 10].

Plain packaging is an element of health warnings in the TCS, and In March 2015, UK Parliament approved plain packaging to begin in May 2016 [31]. Plain packaging has been shown to increase quitting intentions and other quitting-related behaviours among smokers [32]. Following implementation of plain packaging in Australia, there was a doubling in the percent of smokers who notice the warnings first, and who do not like the look of their packs [33]. We therefore assumed that plain packaging itself would be double the maximum effectiveness of health warnings alone (2 % prevalence reduction only from plain packaging, on top of additional improvements by increasing the size of current health warnings). Some experts have predicted about 1 % absolute prevalence reductions [34], which would correspond to about 5 % relative reduction. We chose to use the more conservative 2 % relative reduction due to the uncertainty around the use of expert elicitation. Support for plain packs in Australia was observed to have no SEC gradient [35].

#### Treatment

UK has most treatment elements in place (recording smoking status, brief advice, quit-line, network cessation support), and is only lacking full reimbursement. Maximum effectiveness was based on other modelling studies [21, 23], and only a small improvement was assumed possible. Smoking cessation services in England have produced essentially constant success rates across SEC [36].

Each policy improvement would result in a proportional decline in smoking prevalence. For example, a 10 % decline from a baseline of 20 % smoking prevalence would mean a 2 % absolute decline. The potential reductions in smoking prevalence for each policy type are in Table 1. Where policies are known to have a socioeconomic gradient in effectiveness, we modified the SEC-specific effectiveness accordingly.

### Health outcomes

To illustrate health improvement associated with reduced smoking prevalence for 2015–2025, we first forecasted CHD mortality by 10-year age groups, sex and IMDQ up to 2025, using a Bayesian age-period-cohort model [37, 38]. Then, we translated the modelled reduction in smoking prevalence into reduction of the forecasted number of deaths, through a reduction in the population attributable risk fraction for smoking. For a more detailed description, please refer to the Additional file 1. A slow, steady reduction in smoking prevalence, as was previously observed in England [2], is considered in our estimations. This is because the forecast of CHD deaths is based on previous recent trends of CHD related risk factors, including smoking. We report premature (ages 35–74) CHD deaths prevented or postponed (DPP) and the associated life years gained (LYG) for 2015–2025, stratified by sex and IMDQ.

## Results

Improving all smoking policies to maximize the TCS could reduce overall smoking prevalence in England from 20 % to approximately 17 % (95 % Confidence Interval (CI): 16.0–18.7 %). This would represent an absolute decrease of some 3 % and a relative reduction of approximately 15 % (95 % CI: 7–21 %). Table 2 shows the adult smoking prevalence for England by IMDQ and gender, at baseline and with full implementation of the TCS policies.

**Table 2:** Smoking prevalence at baseline (2012 ONS data) and with all Tobacco Control Scale policies maximised

IMDQ	Sex	Smoking prevalence					Premature CHD deaths			Life years gained	
		Baseline	With policies	95 % CI	Relative Reduction	95 % CI	Baseline	Reduction	95 % CI		95 % CI
1	Men	13.1 %	11.8 %	(10.4–12.4 %)	10 %	(6–20 %)	16100	180	(130–280)	2800	(2000–4300)
2	Men	16.7 %	14.7 %	(13.3–15.5 %)	12 %	(7–20 %)	20900	290	(210–390)	4500	(3300–6100)
3	Men	21.1 %	18.2 %	(16.9–19.5 %)	14 %	(7–20 %)	25300	440	(310–620)	6700	(4800–9500)
4	Men	25.6 %	21.8 %	(20.3–23.5 %)	15 %	(8–20 %)	28700	630	(440–900)	9300	(6600–13300)
5	Men	34.3 %	27.9 %	(27.0–31.8 %)	19 %	(7–21 %)	32600	900	(610–1220)	12800	(8800–17200)
1	Women	10.2 %	9.2 %	(8.1–9.6 %)	10 %	(6–20 %)	4100	50	(30–80)	900	(600–1400)
2	Women	13.5 %	11.9 %	(10.8–12.6 %)	12 %	(7–20 %)	5300	80	(60–110)	1400	(1000–2000)
3	Women	17.0 %	14.6 %	(13.6–15.7 %)	14 %	(7–20 %)	6900	130	(80–190)	2300	(1500–3500)
4	Women	21.4 %	18.2 %	(17.0–19.7 %)	15 %	(8–20 %)	10300	250	(150–400)	4300	(2700–7100)
5	Women	28.3 %	23.0 %	(22.2–26.2 %)	19 %	(7–21 %)	12500	370	(210–680)	6500	(3700–11900)

Premature (ages 35–74) coronary heart disease (CHD) deaths and reduction with policies implemented, aggregate on 2015–2025. Calculations are described in Additional file 1. 95 % confidence intervals (CI) from probabilistic sensitivity analysis of key parameters. Results stratified by sex and quintile groups of Index of Multiple Deprivation (IMDQ, 1 = least deprived, 5 = most deprived)

**Fig. 1:**
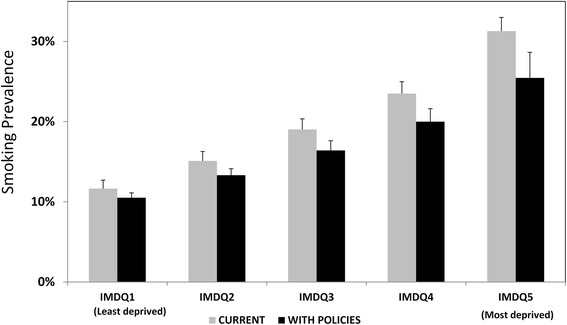
Observed vs. estimated smoking prevalence after maximising the Tobacco Control Scale. Stratified by quintiles of Index of Multiple Deprivation (IMDQ), for ages 35–74, England. Average smoking prevalence for IMDQ is a weighted average across ages 35–74 using the European Standard. These weighted averages for men and women are themselves averaged at the IMDQ level. Error bars are 95 % confidence intervals based on probabilistic sensitivity analysis

The effect would be greatest among the most deprived quintile, who might achieve an absolute reduction of approximately 5.8 % (95 % CI: 2.2–6.7 %) and a relative reduction of 19 % (95 % CI: 7–21 %) (Fig. 1, Table 2). The reduction would be higher in men (6.4 %; 95 % CI: 2.8–6.9 %), because they have higher baseline smoking prevalence, than in women (5.3 %; 95 % CI: 2.2–5.8 %).

Taxes might contribute about 48 % (95 % CI: 41–57 %) of the predicted decline, and this effect could be substantially larger in the most deprived quintile (68 %). Plain packs would contribute about 15 % overall (95 % CI: 11–17 %) (Additional file 1: Table S3).

The model estimated that a total of approximately 3300 (95 % CI: 2200–4700) premature CHD deaths might be prevented or postponed, about 2 % (95 % CI: 1.4–2.9 %) of all predicted premature CHD deaths from 2015–2025 (Table 2). These deaths prevented or postponed would lead to approximately 52,000 LYG (95 % CI: 35,000–76,000). Due to their higher smoking prevalence and higher CHD mortality, the most deprived quintile should benefit more, resulting in an absolute inequality reduction of some 1000 (95 % CI: 700–2200) premature CHD deaths. The most deprived quintile would also gain the most life years (19,000; 95 % CI: 13,000–29,000). Even if there is no SEC gradient in the tax policy effect (Additional file 1: Table S4), the benefits would still favour the most disadvantaged groups, but the results would not be as strong.

Among men, most (about two-thirds) of the life years gained would be in the age groups 45–54 and 55–64. The remainder would be about evenly split among 35–44 and 65–74. This demonstrates that among younger men even a small reduction in CHD mortality can lead to a substantial gain in life years. This holds across all deprivation quintiles. Among women, most of the gain in life years is at ages 55–64 and 65–74. The CHD mortality at ages 35–44 and 45–54 is too low among women for there to be substantial improvement except among the most deprived (Table 3).

**Table 3:** Absolute reduction in smoking prevalence, CHD deaths prevented or postponed (DPP), and Life years gained (LYG)

			Absolute reduction in smoking prevalence	CHD DPP	LYG from DPP
Men	IMDQ1	Ages 35–44	2.0 % (0.9–3.3 %)	10 (5–20)	400 (200–600)
		Ages 45–54	1.8 % (0.8–2.9 %)	40 (30–60)	800 (600–1300)
		Ages 55–64	1.4 % (0.5–2.5 %)	70 (50–110)	1100 (800–1600)
		Ages 65–74	1.0 % (0.1–2.0 %)	60 (40–90)	300 (200–500)
	IMDQ2	Ages 35–44	2.9 % (1.7–4.2 %)	20 (10–30)	700 (400–1000)
		Ages 45–54	2.3 % (1.2–3.4 %)	60 (50–80)	1300 (1000–1800)
		Ages 55–64	1.8 % (0.7–2.9 %)	110 (80–140)	1600 (1200–2100)
		Ages 65–74	1.3 % (0.3–2.4 %)	100 (70–140)	600 (400–800)
	IMDQ3	Ages 35–44	3.5 % (2.2–4.8 %)	30 (20–40)	800 (500–1200)
		Ages 45–54	3.0 % (1.8–4.3 %)	90 (70–130)	2000 (1400–2700)
		Ages 55–64	2.7 % (1.4–4.0 %)	190 (130–260)	2700 (1900–3700)
		Ages 65–74	1.5 % (0.4–2.6 %)	130 (90–190)	800 (500–1200)
	IMDQ4	Ages 35–44	4.3 % (2.7–5.6 %)	40 (20–50)	1100 (700–1700)
		Ages 45–54	3.9 % (2.4–5.3 %)	130 (100–170)	2800 (2000–3700)
		Ages 55–64	3.2 % (1.6–4.6 %)	240 (180–340)	3500 (2500–4800)
		Ages 65–74	2.6 % (0.9–4.1 %)	220 (150–310)	1500 (900–2400)
	IMDQ5	Ages 35–44	5.5 % (3.3–7.2 %)	50 (30–70)	1500 (900–2100)
		Ages 45–54	5.4 % (3.2–7.2 %)	180 (130–240)	3800 (2600–4900)
		Ages 55–64	4.9 % (2.6–6.8 %)	360 (250–470)	4900 (3400–6400)
		Ages 65–74	3.7 % (1.5–5.6 %)	300 (200–420)	2000 (1200–3500)
Women	IMDQ1	Ages 35–44	1.5 % (0.5–2.5 %)	1 (0–2)	30 (0–80)
		Ages 45–54	1.4 % (0.5–2.3 %)	6 (4–9)	160 (100–240)
		Ages 55–64	1.2 % (0.3–2.2 %)	20 (10–30)	340 (230–520)
		Ages 65–74	0.9 % (0.1–1.8 %)	25 (20–40)	600 (400–800)
	IMDQ2	Ages 35–44	2.1 % (1.0–3.1 %)	1 (0–3)	50 (0–100)
		Ages 45–54	1.9 % (0.9–2.8 %)	8 (6–12)	230 (170–340)
		Ages 55–64	1.7 % (0.7–2.8 %)	30 (20–40)	600 (400–800)
		Ages 65–74	1.1 % (0.2–2.0 %)	40 (30–60)	900 (600–1300)
	IMDQ3	Ages 35–44	2.7 % (1.5–3.8 %)	3(0–7)	100 (0–300)
		Ages 45–54	2.5 % (1.4–3.6 %)	15(10–25)	400 (300–600)
		Ages 55–64	2.3 % (1.1–3.4 %)	50(30–70)	900 (600–1300)
		Ages 65–74	1.4 % (0.4–2.4 %)	60(40–90)	1200 (800–1700)
	IMDQ4	Ages 35–44	3.3 % (1.9–4.5 %)	6 (0–13)	200 (0–500)
		Ages 45–54	3.4 % (1.9–4.6 %)	30 (20–50)	900 (500–1400)
		Ages 55–64	2.9 % (1.4–4.2 %)	90 (60–140)	1700 (1100–2700)
		Ages 65–74	2.1 % (0.7–3.5 %)	120 (80–190)	1900 (1300–2700)
	IMDQ5	Ages 35–44	4.2 % (2.3–5.8 %)	10 (0–30)	500 (0–1000)
		Ages 45–54	4.6 % (2.6–6.3 %)	60 (40–100)	1600 (900–2700)
		Ages 55–64	4.1 % (2.0–5.8 %)	130 (80–220)	2500 (1400–4200)
		Ages 65–74	3.2 % (1.2–4.9 %)	170 (100–290)	2600 (1700–3600)

Stratified by age group, sex, and quintiles of Index of Multiple Deprivation (IMDQ, 1 = least deprived, 5 = most deprived). 95 % confidence intervals from probabilistic sensitivity analysis

## Discussion

We modelled the effect that fully implementing all elements of the TCS would have on smoking prevalence in England and how the resultant changes might vary by socioeconomic circumstance. Improving all smoking policies to achieve a maximum score on the TCS might reduce smoking prevalence in England by approximately 15 % in relative terms among adults ages 35–74, and even more in the most deprived socioeconomic quintiles. There would be over 3000 fewer premature CHD deaths with the biggest benefits in the most deprived groups.

Reassuringly, our estimated smoking prevalence reduction attributable to the modelled tobacco control policies seems similar to estimates from a recent study using SimSmoke (~15 % versus ~17 % of relative reduction) [39]. Unfortunately, direct comparison of IMPACT and SimSmoke on avoidable deaths is not possible, because SimSmoke estimates deaths from any smoke-attributed disease rather than CHD specifically.

We estimated that an excise tax increase that increased pack price by 20 % would contribute about half of the total reduction in smoking prevalence. This increase is feasible, considering the price of tobacco has increased by more than 80 % over the last ten years [2]. Excise tax increases are generally considered the most immediate and effective path to quitting [40]. In reality, evidence suggest that the more deprived are more sensitive to price change and reduce consumption more than the less deprived [18–20], rendering tobacco price policies the most equitable option for tobacco control [12]. In addition, the increased tax revenue may be redistributed to the less well-off through targeted smoking cessation and other health promotion programmes, further increasing the equity of this policy.

The TCS is based on a comprehensive, multi-pronged approach to tobacco control and measures three key areas: accessibility, affordability, and acceptability. It is generally agreed that comprehensive approaches to tobacco control work best because they lower initiation, increase cessation and reduce consumption [8, 41]. The TCS score across countries has been shown to be positively correlated with quit attempts [42]. TCS is therefore a useful metric when focussing on adult smoking prevalence, as we have done. Most of the policy categories would also counteract youth initiation of smoking, but different effect sizes and SEC gradients might be needed.

Despite the usefulness of TCS score for policy analysis and relative intra-country comparisons a TCS of 100 does not mean that there is no further room for improvement. This is especially true for countries already scoring high in the TCS, like UK. A typical example is policies on tobacco price, where UK has the highest in Europe; yet it can increase even further depending on political will, achieving a score higher than 100. Therefore, our decision to model the optimum TCS score is based on the possible usefulness for policy makers rather than an ultimate goal for UK tobacco control policy.

Stricter smoking policies have already contributed to rapid improvements in cardiovascular health in the UK [43, 44] and have reduced health inequalities in Ireland [45]. CHD and other vascular diseases can show rapid improvements in mortality due to risk factor changes [46]. We demonstrated that declines in smoking prevalence would lower premature CHD mortality and that the most disadvantaged socioeconomic groups would benefit most. This would reduce absolute inequality of premature CHD mortality. Many other tobacco related cancers and other non-communicable diseases would also benefit from smoking cessation, though the time lag between risk factor change and mortality improvement may not be as immediate.

Improvements in the policies we assessed would make a dent in current adult smoking prevalence in England, but further policy initiatives could have additional impacts and would be required to begin talk of a “Tobacco End Game” [47, 48]. Recent research suggests that e-cigarettes might help with smoking cessation in the short-term but not in the long-term, although further research is needed [49, 50]. A ban on smoking in cars due to be implemented in England on 1^st^ October 2015 could lead to more people declaring their homes smoke-free [51], which itself might enable quit attempts [52]. Small trials of financial incentives for pregnant women to stop smoking appear promising [53]. The government has also recently consulted on the possibility of a minimum excise tax and a levy on tobacco companies [54], both of which could raise prices further and, in the case of the former, help narrow inequalities in smoking by closing the price gap between cheap and expensive cigarettes [55]. When the tobacco purchase age was raised from 16 to 18 in England, smoking prevalence declined dramatically among 16–17 year olds [56]. Further raising the tobacco purchase age, as currently in place or under consideration in parts of the US, might accelerate long-term reductions [57, 58]. Finally, various more innovative options including ‘very low nicotine content’ (VLNC) cigarettes sold at a lower price than standard cigarettes could play a role in further reducing tobacco use [59]. Yet, challenges lay ahead. The observed and forthcoming reductions in funding for mass media campaigns and smoking cessation services may have detrimental effects on tobacco control and postpone the ‘end game’ further in the future [25, 60].

### Strengths

Modelling studies of this type are rarely stratified by socioeconomic circumstance, and none has yet done so for England. We used four key systematic reviews [9–12] to derive information about SEC gradients in effectiveness of tobacco control policies. We reviewed many of the studies cited by these four systematic reviews and actively checked for newer studies.

We linked declines in smoking prevalence to declines in CHD mortality, using a model validated by SEC for England [14]. The smoking prevalence reported by ONS was stratified into the same strata (IMDQ, age, sex) as the CHD mortality, thus no additional assumptions were necessary to link the two.

### Limitations

We focussed only on adult smoking prevalence, reductions in CHD and used a short time-frame. The long-term effects might be about twice as large when considering reduced youth initiation [39] and greater still, when considering other tobacco related diseases and environmental tobacco smoking.

Despite our effort to identify the best available sources to inform our model, there is a lack of strong evidence regarding the existence and quantification of the socioeconomic gradient for the effect of some of the modelled policies. This is also true for the combined effect of the interventions. We assumed multiplicative effects of the combined modelled policies, given the lack of evidence to support any functional form for the combined effect.

Moreover, in our study we ignored the rise in e-cigarettes use, a potentially important emerging trend. Unfortunately, none of the data sources used to inform IMPACT considered e-cigarettes specifically, and their overall impact on smokers and non-smokers is lacking consistent evidence.

Finally, we did not consider implementation. Now that many public health functions in England have a stronger local element than before, there is greater likelihood of inconsistent implementation.

## Conclusions

A comprehensive improvement in tobacco control policies in England could have immediate, long-lasting effects. Health would improve substantially more among those who stand to gain the most, thus narrowing health-related inequalities. Further research is needed to explore the equity of specific tobacco control policies and to identify their optimal mixture to maximise efficiency and equity; particularly considering the challenges that ongoing cuts in Public Health funding across England pose to communities.

## Additional file

Additional file 1: Technical Appendix. (DOCX 36 kb)

## References

[1] Opinions and lifestyle survey, adult smoking habits in Great Britain, 2013. [http://www.ons.gov.uk/ons/rel/ghs/opinions-and-lifestyle-survey/adult-smoking-habits-in-great-britain--2013/index.html]. Accessed 03 Aug 2015.

[2] Statistics on smoking: England 2014 [http://www.hscic.gov.uk/catalogue/PUB14988].Accessed 03 Aug 2015.

[3] Hiscock R, Bauld L, Amos A, Platt S (2012). Smoking and socioeconomic status in England: the rise of the never smoker and the disadvantaged smoker. J Public Health Oxf Engl.

[4] Giesinger I, Goldblatt P, Howden-Chapman P, Marmot M, Kuh D, Brunner E (2014). Association of socioeconomic position with smoking and mortality: the contribution of early life circumstances in the 1946 birth cohort. J Epidemiol Community Health.

[5] Action on Smoking and Health (ASH) (2014). Smoking statistics: illness and death.

[6] Nabi H, Estaqiuo C, Auleley G-R (2015). Smoking and mortality--beyond established causes. N Engl J Med.

[7] Joossens L, Raw M (2014). The tobacco control scale 2013 in Europe. Sixth European Conference on Tobacco or Health-ECToH, Istanbul, Turkey.

[8] World Health Organization, others (2013). WHO Report on the Global Tobacco Epidemic, 2013: Enforcing Bans on Tobacco Advertising, Promotion and Sponsorship.

[9] Thomas S, Fayter D, Misso K, Ogilvie D, Petticrew M, Sowden A, Whitehead M, Worthy G (2008). Population tobacco control interventions and their effects on social inequalities in smoking: systematic review. Tob Control.

[10] Hill S, Amos A, Clifford D, Platt S (2014). Impact of tobacco control interventions on socioeconomic inequalities in smoking: review of the evidence. Tob Control.

[11] Brown T, Platt S, Amos A (2014). Equity impact of European individual-level smoking cessation interventions to reduce smoking in adults: a systematic review. Eur J Public Health.

[12] Brown T, Platt S, Amos A (2014). Equity impact of population-level interventions and policies to reduce smoking in adults: A systematic review. Drug Alcohol Depend.

[13] Unal B, Critchley JA, Capewell S (2004). Explaining the decline in coronary heart disease mortality in England and Wales between 1981 and 2000. Circulation.

[14] Bajekal M, Scholes S, Love H, Hawkins N, O’Flaherty M, Raine R, Capewell S (2012). Analysing recent socioeconomic trends in coronary heart disease mortality in England, 2000–2007: a population modelling study. PLoS Med.

[15] Bandosz P, Aspelund T, Basak P, Bennett K, Bjorck L, Bruthans J, Guzman-Castillo M, Hughes J, Hotchkiss J, Kabir Z, Laatikainen T, Leyland A, O’Flaherty M, Palmieri L, Rosengren A, Bjork R, Vartiainen E, Zdrojewski T, Capewell S, Critchley J (2014). OP72 EUROHEART II - comparing policies to reduce future coronary heart disease mortality in nine European countries: modelling study. J Epidemiol Community Health.

[16] Do smoking rates vary between more and less advantaged areas? [http://www.ons.gov.uk/ons/rel/disability-and-health-measurement/do-smoking-rates-vary-between-more-and-less-advantaged-areas-/2012/sty-smoking-rates.html]. Accessed 03 Aug 2015

[17] McLennan D, Barnes H, Noble M, Davies J, Garratt E, Dibben C (2011). The English Indices of Deprivation 2010.

[18] Townsend J (1996). Price and consumption of tobacco. Br Med Bull.

[19] Reed H (2010). The Effects of Increasing Tobacco Taxation: A Cost Benefit and Public Finances Analysis.

[20] International Agency for Research on Cancer (2011). IARC Handbooks of Cancer Prevention, Tobacco Control, Vol. 14: Effectiveness of Tax and Price Policies for Tobacco Control.

[21] Levy DT, Currie L, Clancy L (2013). Tobacco control policy in the UK: blueprint for the rest of Europe?. Eur J Public Health.

[22] Nagelhout GE, Levy DT, Blackman K, Currie L, Clancy L, Willemsen MC (2012). The effect of tobacco control policies on smoking prevalence and smoking-attributable deaths. Findings from the Netherlands SimSmoke Tobacco Control Policy Simulation Model. Addict Abingdon Engl.

[23] Fichtenberg CM, Glantz SA (2002). Effect of smoke-free workplaces on smoking behaviour: systematic review. BMJ.

[24] Langley T, Szatkowski L, Lewis S, McNeill A, Gilmore AB, Salway R, Sims M (2014). The freeze on mass media campaigns in England: a natural experiment of the impact of tobacco control campaigns on quitting behaviour. Addict Abingdon Engl.

[25] Action on Smoking and Health (ASH) (2014). ASH Briefing: UK tobacco control policy and expenditure.

[26] Levy DT, Friend K (2001). A computer simulation model of mass media interventions directed at tobacco use. Prev Med.

[27] Sims M, Salway R, Langley T, Lewis S, McNeill A, Szatkowski L, Gilmore AB (2014). Effectiveness of tobacco control television advertising in changing tobacco use in England: a population-based cross-sectional study. Addict Abingdon Engl.

[28] Niederdeppe J, Kuang X, Crock B, Skelton A (2008). Media campaigns to promote smoking cessation among socioeconomically disadvantaged populations: what do we know, what do we need to learn, and what should we do now?. Soc Sci Med 1982.

[29] Saffer H, Chaloupka F (1999). Tobacco Advertising: Economic Theory and International Evidence, Working Paper.

[30] Chaloupka FJ, Warner KE, Acemoğlu D, Gruber J, Laux F, Max W, Newhouse J, Schelling T, Sindelar J (2015). An evaluation of the FDA’s analysis of the costs and benefits of the graphic warning label regulation. Tob Control.

[31] UK first EU country to adopt plain packaging for cigarettes [http://www.euractiv.com/sections/health-consumers/uk-first-eu-country-adopt-plain-packaging-cigarettes-312960]. Accessed 03 Aug 2015

[32] Durkin S, Brennan E, Coomber K, Zacher M, Scollo M, Wakefield M (2015). Short-term changes in quitting-related cognitions and behaviours after the implementation of plain packaging with larger health warnings: findings from a national cohort study with Australian adult smokers. Tob Control.

[33] British Heart Foundation (2014). Standardised Packaging for Tobacco Products: Recent Evidence from Australia and United Kingdom.

[34] Pechey R, Spiegelhalter D, Marteau TM (2013). Impact of plain packaging of tobacco products on smoking in adults and children: an elicitation of international experts’ estimates. BMC Public Health.

[35] Swift E, Borland R, Cummings KM, Fong GT, McNeill A, Hammond D, Thrasher JF, Partos TR, Yong H-H (2014). Australian smokers’ support for plain or standardised packs before and after implementation: findings from the ITC Four Country Survey. Tob Control..

[36] Bauld L, Judge K, Platt S (2007). Assessing the impact of smoking cessation services on reducing health inequalities in England: observational study. Tob Control.

[37] Guzman Castillo M, Gillespie DOS, Allen K, Bandosz P, Schmid V, Capewell S, O’Flaherty M (2014). Future declines of coronary heart disease mortality in England and Wales could counter the burden of population ageing. PloS One.

[38] Schmid VJ, Held L (2007). Bayesian age-period-cohort modeling and prediction-BAMP. J Stat Softw.

[39] Levy DT, Huang A-T, Currie LM, Clancy L (2014). The benefits from complying with the framework convention on tobacco control: a SimSmoke analysis of 15 European nations. Health Policy Plan.

[40] Jha P, Peto R (2014). Global effects of smoking, of quitting, and of taxing tobacco. N Engl J Med.

[41] Centers for Disease Control, (CDC) P, others (2014). Best Practices for Comprehensive Tobacco Control programs—2014.

[42] Schaap MM, Kunst AE, Leinsalu M, Regidor E, Ekholm O, Dzurova D, Helmert U, Klumbiene J, Santana P, Mackenbach JP (2008). Effect of nationwide tobacco control policies on smoking cessation in high and low educated groups in 18 European countries. Tob Control.

[43] Pell JP, Haw S, Cobbe S, Newby DE, Pell ACH, Fischbacher C, McConnachie A, Pringle S, Murdoch D, Dunn F, Oldroyd K, Macintyre P, O’Rourke B, Borland W (2008). Smoke-free legislation and hospitalizations for acute coronary syndrome. N Engl J Med.

[44] Sims M, Maxwell R, Bauld L, Gilmore A (2010). Short term impact of smoke-free legislation in England: retrospective analysis of hospital admissions for myocardial infarction. BMJ.

[45] Stallings-Smith S, Goodman P, Kabir Z, Clancy L, Zeka A (2014). Socioeconomic differentials in the immediate mortality effects of the national Irish smoking ban. PloS One.

[46] Morita H, Ikeda H, Haramaki N, Eguchi H, Imaizumi T (2005). Only two-week smoking cessation improves platelet aggregability and intraplatelet redox imbalance of long-term smokers. J Am Coll Cardiol.

[47] van der Eijk Y (2015). Development of an integrated tobacco endgame strategy. Tob Control.

[48] Warner KE (2013). An endgame for tobacco?. Tob Control.

[49] Brose LS, Hitchman SC, Brown J, West R, McNeill A (2015). Is the use of electronic cigarettes while smoking associated with smoking cessation attempts, cessation and reduced cigarette consumption? A survey with a 1-year follow-up. Addict Abingdon Engl.

[50] Al-Delaimy WK, Myers MG, Leas EC, Strong DR, Hofstetter CR (2015). E-cigarette use in the past and quitting behavior in the future: a population-based study. Am J Public Health.

[51] Murphy-Hoefer R, Madden P, Maines D, Coles C (2014). Prevalence of smoke-free car and home rules in Maine before and after passage of a smoke-free vehicle law, 2007–2010. Prev Chronic Dis.

[52] Borland R, Yong H-H, Cummings KM, Hyland A, Anderson S, Fong GT (2006). Determinants and consequences of smoke-free homes: findings from the International Tobacco Control (ITC) Four Country Survey. Tob Control.

[53] Tappin D, Bauld L, Purves D, Boyd K, Sinclair L, MacAskill S, McKell J, Friel B, McConnachie A, de Caestecker L, Tannahill C, Radley A, Coleman T, Cessation in Pregnancy Incentives Trial Team (2015). Financial incentives for smoking cessation in pregnancy: randomised controlled trial. BMJ.

[54] Minimum excise tax [https://www.gov.uk/government/consultations/minimum-excise-tax/minimum-excise-tax]. Accessed 03 Aug 2015.

[55] Gilmore AB, Tavakoly B, Taylor G, Reed H (2013). Understanding tobacco industry pricing strategy and whether it undermines tobacco tax policy: the example of the UK cigarette market. Addict Abingdon Engl.

[56] Fidler JA, West R (2010). Changes in smoking prevalence in 16-17-year-old versus older adults following a rise in legal age of sale: findings from an English population study. Addict Abingdon Engl.

[57] Steinberg MB, Delnevo CD (2013). Increasing the “smoking age”: the right thing to do. Ann Intern Med.

[58] Bonnie RJ, Alberg AJ, Nola RB, Caulkins J, Halpern-Felsher B, Jett S, Juster H, Klein JD, Lantz PM, Mermelstein R, Meza R, O’Malley P, Thompson K (2015). Public Health Implications of Rising the Minimum Age of Legal Access to Tobacco Products.

[59] Walker N, Fraser T, Howe C, Laugesen M, Truman P, Parag V, Glover M, Bullen C (2014). Abrupt nicotine reduction as an endgame policy: a randomised trial. Tob Control..

[60] Lacobucci G (2016). Public health—the frontline cuts begin. BMJ.

