# Supplementary material for: The effects of maximising the UK’s tobacco control score on inequalities in smoking prevalence and premature coronary heart disease mortality: a modelling study
Source: BMC Public Health. 2016 Apr 1;16:292. doi: 10.1186/s12889-016-2962-8 (PMC4818400; doi:10.1186/s12889-016-2962-8)
Supplement: Additional file 1: — Technical Appendix. (DOCX 36 kb) [file 12889_2016_2962_MOESM1_ESM.docx]

TECHNICAL APPENDIX

# Population projections

Office for National Statistics (ONS) published population counts by age group, sex, and quintile groups of Index Of Multiple Deprivation (IMDQ) for 2002-2013. Unfortunately, ONS does not publish population projections by IMDQ, neither fertility rates by IMDQ. To overcome this, we assumed that the distribution of population within IMDQ up to 2015, will remain as of 2013 by age group and sex. Thus, we first summed the five-year age bands into ten-year age bands, by sex. For each age-sex group, we calculated the proportion of the population in 2013 in each IMDQ. We then applied these proportions to population projections from 2015-2025. An example follows for men aged 65-74:

| IMDQ | M 65 - 69 | M 70 - 74 | M 65 – 74 (Sum) | Proportion |
| --- | --- | --- | --- | --- |
| 5 | 200,601 | 146,679 | 347,280 | 0.144 |
| 4 | 242,979 | 173,038 | 416,017 | 0.172 |
| 3 | 303,520 | 214,651 | 518,171 | 0.214 |
| 2 | 337,354 | 235,710 | 573,064 | 0.237 |
| 1 | 335,226 | 228,845 | 564,071 | 0.233 |
| Total Sum |  |  | 2,418,603 | 1 |

Then, based on ONS constant fertility population projections for England, we created the relevant age groups for each sex and multiplied by the IMDQ proportions from 2013. Continuing the example:

| Year |  | 2015 | 2016 | 2017 | … | 2025 |
| --- | --- | --- | --- | --- | --- | --- |
| Projected M 65-74 (ONS) |  | 2,541,495 | 2,602,704 | 2,642,573 | … | 2,733,938 |
| Proportion estimates by IMDQ (Our calculations, see above) | |  |  |  |  |  |
| 5 | 0.144 | 364,926 | 373,715 | 379,439 | … | 392,558 |
| 4 | 0.172 | 437,155 | 447,684 | 454,541 | … | 470,257 |
| 3 | 0.214 | 544,500 | 557,614 | 566,155 | … | 585,730 |
| 2 | 0.237 | 602,182 | 616,685 | 626,131 | … | 647,780 |
| 1 | 0.233 | 592,732 | 607,007 | 616,306 | … | 637,614 |

## Data sources

For the calculation above we used the data sources below:

“Number of deaths and populations broken down by sex, year, the adjusted IMD 2010 quintiles of English Lower Super Output Areas and age group, 2002-2013”. Available with reference number 003497 at <http://www.ons.gov.uk/ons/about-ons/business-transparency/freedom-of-information/what-can-i-request/published-ad-hoc-data/health/november-2014/index.html> [26 November 2014]

“National Population Projections, 2012-based extra variants”. Available from: <http://www.ons.gov.uk/ons/rel/npp/national-population-projections/2012-based-extra-variants/index.html> ; Specifically, the following ZIP file [caution: might download automatically] : <http://www.ons.gov.uk/ons/rel/npp/national-population-projections/2012-based-extra-variants/rft-table-z8-zipped-population-data-files---england.zip>

# Health Outcomes

Analyses were stratified by age group (10-year bands from 35-44 to 65-74), sex, and socioeconomic circumstance. Ages above 75 were not modelled because smoking is rare beyond this age [1] and because premature mortality (< 75) has stronger socioeconomic gradients than total mortality [2]. For socioeconomic circumstance, we used quintiles of the Index of Multiple Deprivation (IMDQ) [3] based on Public Health England’s 2010 adjusted IMD [4].

For the time horizon 2015 - 25, baseline CHD mortality was estimated based on past trends from 2002 - 2013 (ONS ad hoc data request) by age, sex, and IMDQ using an age-period-cohort model [5, 6]. Reductions in premature CHD mortality associated with changes in smoking prevalence were estimated using the population attributable risk fraction approach [7] (example in following sub-section). Changes in premature CHD mortality were converted to absolute numbers of deaths prevented or postponed (DPP) using population projections from ONS, as described above.

The Slope Index [8] was used to measure the inequality of premature CHD deaths. The change in slope index is based on the aggregate numbers of deaths by IMDQ under the baseline scenario compared to the scenario with policies implemented.

We also calculated Life Years Gained (LYG) using a method validated for CHD [9].

## Population attributable risk fraction (PARF) approach

The CHD mortality risk ratios for smoking from the 2001 Global Burden of Disease Study [10] were adapted to the age groups used in the IMPACT-SEC model [7].

The population attribute risk fraction (PARF) calculates the proportion by which CHD mortality would be reduced if there were no smoking in the population. Symbolically, PARF is the following:

PARF_ijk_ = [P_ijk_ × (RR_ij_ - 1)] / [1 + P_ijk_ × (RR_ij_ - 1)]

where P_ijk_ is the smoking prevalence for age *i*, sex *j*, and IMDQ *k*

RR_ij_ is the CHD risk ratio for age *i* and sex *j* (assumed not to vary by IMDQ)

For example, if smoking prevalence is 20% and the risk ratio is 2.0, PARF is 17%. This means 17% of CHD deaths are attributable to smoking. We are interested in how this PARF changes as a result of changes in smoking prevalence. Symbolically, this would be:

ΔPARF_ijk_ = PARF_ijk_ – PARF’_ijk_

where PARF’ is the calculated for the (lower) smoking prevalence after a policy change

As defined here, the change in PARF (ΔPARF_ijk_) is positive when the smoking prevalence declines. For the combined effect of all policies, we used a multiplicative effect. For example, if two policies each lower prevalence by 5%, the multiplicative effect is 9.75% [1 - (1 - 0.05)*(1 - 0.05)] rather than a simple addition of 10% [0.05 + 0.05].

The ΔPARF is then multiplied by the CHD mortality counterfactual and population projection to obtain the CHD DPP:

DPP_ijk_ = Pop_ijk_ × Mort_ijk_ × ΔPARF_ijk_

# Sensitivity Analysis

Probabilistic sensitivity analysis was performed to account for uncertainty in modelled parameters. Simulation was performed in R version 3.0.1 using 1,000 iterations. The uncertainty and probability distributions used are shown in Table S1

# Tables

Table S 1 Model parameters and uncertainty. 1,000 simulations were performed in R version 3.0.1 with the probability distributions listed

| PARAMETER | CENTRAL ESTIMATE | UNCERTAINTY |
| --- | --- | --- |
| Smoking prevalence in 2012 | From ONS [12] | Sample sizes by age and sex pulled from Integrated Household Survey. Split into IMDQ using method described above. Normal approximation to binomial assumed. |
| Policy effect sizes | See Table 1 | Assume PERT distribution [11] with 20% uncertainty around given values |
| Policy SEC gradients | See Table 1 | Probability of gradient direction proportional to number of studies from [12, 13]. |
| CHD Risk Ratios for smoking | Adapted from Global Burden of Disease 2001 [7, 10] | Normal distribution based on lower and upper confidence intervals |
| CHD mortality for 2015-2025 | Extrapolated from data on 2002-2013 using an age-period-cohort model [2] | Normal distribution of the *logit* of rates from model, based on lower and upper credible intervals [6] |

Table S 2 Price elasticity by quintile group of Index of Multiple Deprivation (IMDQ)

| IMDQ | ELASTICITY |
| --- | --- |
| 1 (least deprived) | 0.12 |
| 2 | 0.24 |
| 3 | 0.35 |
| 4 | 0.42 |
| 5 (most deprived) | 0.63 |

Table S 3 Contribution of the different policy components to the modelled prevalence reduction. Brackets contain 95% confidence intervals

| POLICY | CONTRIBUTION (95% confidence intervals) |
| --- | --- |
| Price | 48% (41% - 57%) |
| Smoke-free places | 7% (6% - 8%) |
| Public information campaigns | 7% (6% - 8%) |
| Advertising bans | 15% (12% - 16%) |
| Health Warnings | 7% (6% - 8%) |
| Plain packaging | 15% (12% - 16%) |
| Treatment | 4% (3% - 5%) |

Table S 4 Principle results (compare with Table 2 in main text) if all policies are assumed to have no SEC gradient in effectiveness. This means that the relative reduction for all SEC groups is 14%. Since this corresponds to larger absolute reductions in smoking prevalence and because the disadvantaged SEC groups have higher CHD mortality, there would still be substantial gains among those who stand to gain the most. However, the differences across the SEC groups would be less than calculated in the principle analysis.

| IMDQ | SEX | SMOKING PREVALENCE | | |  |  | PREMATURE CHD DEATHS | | | LIFE YEARS GAINED | |
| --- | --- | --- | --- | --- | --- | --- | --- | --- | --- | --- | --- |
|  |  | Baseline | With policies | 95% CI | Relative Reduction | 95% CI | Baseline | Reduction | 95% CI |  | 95% CI |
| 1 | M | 13.1% | 11.3% | (10.8%-11.7%) | 14% | (11%-18%) | 16100 | 200 | (140-270) | 3100 | (2200 - 4200) |
| 2 | M | 16.7% | 14.4% | (13.8%-14.9%) | 14% | (11%-18%) | 20900 | 300 | (220-390) | 4700 | (3500 - 6000) |
| 3 | M | 21.1% | 18.2% | (17.4%-18.8%) | 14% | (11%-18%) | 25300 | 430 | (300-600) | 6600 | (4700 - 9300) |
| 4 | M | 25.6% | 22.1% | (21.1%-22.8%) | 14% | (11%-18%) | 28700 | 600 | (430-820) | 9000 | (6500 - 12100) |
| 5 | M | 34.3% | 29.6% | (28.3%-30.6%) | 14% | (11%-18%) | 32600 | 840 | (640-1050) | 11800 | (9400 - 14600) |
| 1 | W | 10.2% | 8.8% | (8.4%-9.1%) | 14% | (11%-18%) | 4100 | 50 | (40-70) | 900 | (600 - 1300) |
| 2 | W | 13.5% | 11.7% | (11.1%-12.1%) | 14% | (11%-18%) | 5300 | 80 | (60-110) | 1400 | (1000 - 2000) |
| 3 | W | 17.0% | 14.6% | (14.0%-15.2%) | 14% | (11%-18%) | 6900 | 130 | (80-190) | 2300 | (1500 - 3400) |
| 4 | W | 21.4% | 18.5% | (17.7%-19.1%) | 14% | (11%-18%) | 10300 | 240 | (150-370) | 4100 | (2600 - 6500) |
| 5 | W | 28.3% | 24.4% | (23.3%-25.3%) | 14% | (11%-18%) | 12500 | 350 | (200-560) | 6100 | (3700 - 9900) |
| IMDQ denotes quintile groups of Index of Multiple Deprivation (1 = least deprived, 5 = most deprived)  CI denotes confidence intervals  M denotes Men and W denotes Women | | | | | | | | | | | |

# References

1. **Opinions and lifestyle survey, adult smoking habits in Great Britain, 2013** [http://www.ons.gov.uk/ons/rel/ghs/opinions-and-lifestyle-survey/adult-smoking-habits-in-great-britain--2013/index.html]

2. Allen K, Gillespie DOS, Castillo MG, Diggle PJ, Capewell S, O’Flaherty M: **OP69 Predicting future trends and inequalities in premature coronary heart disease deaths in England: modelling study**. *J Epidemiol Community Health* 2014, **68**(Suppl 1):A35–A35.

3. McLennan D, Barnes H, Noble M, Davies J, Garratt E, Dibben C: *The English Indices of Deprivation 2010*. 2011.

4. **Adjusted IMD 2010 scores for 2011 LSOAs** [http://www.apho.org.uk/resource/item.aspx?RID=125887]

5. Guzman Castillo M, Gillespie DOS, Allen K, Bandosz P, Schmid V, Capewell S, O’Flaherty M: **Future declines of coronary heart disease mortality in England and Wales could counter the burden of population ageing**. *PloS One* 2014, **9**:e99482.

6. Schmid VJ, Held L: **Bayesian age-period-cohort modeling and prediction-BAMP**. *J Stat Softw* 2007, **21**:1–15.

7. Bajekal M, Scholes S, Love H, Hawkins N, O’Flaherty M, Raine R, Capewell S: **Analysing recent socioeconomic trends in coronary heart disease mortality in England, 2000–2007: a population modelling study**. *PLoS Med* 2012, **9**:e1001237.

8. **WHO | Handbook on health inequality monitoring with a special focus on low- and middle-income countries** [http://www.who.int/gho/health_equity/handbook/en/]

9. Smolina K, Wright FL, Rayner M, Goldacre MJ: **Long-term survival and recurrence after acute myocardial infarction in England, 2004 to 2010**. *Circ Cardiovasc Qual Outcomes* 2012, **5**:532–540.

10. *Comparative Quantification of Health Risks*. Geneva: World Health Organisation; 2004. [Ezzati M, Lopez AD, Rodgers A, Murray CJL (Series editors)]

11. Clark CE: **Letter to the Editor-The PERT Model for the Distribution of an Activity Time**. *Oper Res* 1962, **10**:405–406.

12. Thomas S, Fayter D, Misso K, Ogilvie D, Petticrew M, Sowden A, Whitehead M, Worthy G: **Population tobacco control interventions and their effects on social inequalities in smoking: systematic review**. *Tob Control* 2008, **17**:230–237.

13. Hill S, Amos A, Clifford D, Platt S: **Impact of tobacco control interventions on socioeconomic inequalities in smoking: review of the evidence**. *Tob Control* 2014, **23**:e89–97.
